# Educational interventions for erroneous gambling beliefs among Norwegian online casino players: a large-scale real-world study of behavioural impact

**DOI:** 10.1186/s12954-026-01508-9

**Published:** 2026-08-06

**Authors:** Nadia Arshad, Nathan Lakew, Per Carlbring, Ståle Pallesen, Tony Leino, David Forsström, Philip Lindner, Jakob Jonsson

**Affiliations:** 1 Centre for Psychiatry Research, Department of Clinical Neuroscience, Karolinska Institutet & Stockholm Health Care Services, Region Stockholm, Stockholm, Sweden; 2 Department of Global Public Health and Primary Care, Faculty of Medicine, University of Bergen, Bergen, Norway; 3 Department of Psychology, Stockholm University, Stockholm, Sweden; 4 School of Psychology, Korea University, Seoul, South Korea; 5 Department of Psychosocial Science, University of Bergen, Bergen, Norway; 6 Norwegian Competence Center for Gambling and Gaming Research, University of Bergen, Bergen, Norway

**Keywords:** Gambling beliefs, Educational online intervention, Gambling behaviour, Responsible gambling, Online casino

## Abstract

**Background:**

Erroneous beliefs about gambling contribute to the development and maintenance of problem gambling and represent an important public health concern. Norsk Tipping (NT), the state-owned gambling operator in Norway, initiated the Spillepuls project to develop and evaluate preventive interventions for its online customers. This study evaluated an educational intervention that addressed gambling-specific misconceptions among users of NT’s online casino platform. The intervention compared different formats (video versus quiz), delivery modes (voluntary versus mandatory), and timing (pregame versus during play), against a non-intervention control group. The outcome measures comprised gambling behaviour and use of responsible gambling (RG) measures.

**Method:**

The data were collected by NT as part of their routine operations. The experiment took place between May 2022 and January 2023. During this period, NT randomly assigned 18,981 new online casino customers to one of three conditions (Video, Quiz, or Control) on their third day of play. Customers allocated to the Video and Quiz conditions were subsequently randomized to one of three delivery modes: voluntary before gameplay, mandatory before gameplay, or mandatory during gameplay.

**Results:**

No significant differences in gambling behaviour or uptake of RG measures were observed between the intervention groups and the control group. However, comparisons across the six intervention conditions revealed clear differences in engagement. Mandatory pregame delivery produced the highest completion rates, with a small but significant difference between the video and quiz formats (99.8% vs. 98.2%; χ^2^(1, N = 5403) = 10.9, *p* < .01). In contrast, the voluntary pregame video achieved the lowest completion rate (7.1%; χ^2^(1, N = 5435) = 283.4, *p* < .001). Participants rated the video interventions as more relevant and useful than the quizzes in both the mandatory and voluntary conditions, whereas the quiz format was perceived as less intrusive.

**Conclusion:**

Mandatory interventions achieved substantially higher completion rates, whereas voluntary video and quiz interventions were perceived as more relevant and useful by participants. Although mandatory pregame delivery was associated with lower values for selected monetary gambling indicators than voluntary pregame delivery, none of the intervention conditions differed from the control group in gambling behaviour or uptake of RG measures. These findings indicate the need for further research to identify more effective approaches for promoting RG.

**Supplementary Information:**

The online version contains supplementary material available at 10.1186/s12954-026-01508-9.

## Introduction

Gambling, a widely enjoyed leisure activity, involves wagering money (or something of value) on uncertain outcomes in hopes of winning something of greater value [1]. While most engage in gambling without issues, a minority experience gambling-related harms, with some developing disordered gambling [2–4]. Globally, it is estimated that about 46% of adults gambled during the last 12 months, and 1.4% are estimated to be engaged in harmful gambling [5]. In Norway, the setting of this study, the prevalence of gambling is 61.8% and among these, 58.4% had played online at least once. The level of problem gambling is 0.6% and the level of moderate at-risk gambling is 2.3% [6].

Historically, the gambling industry has followed a responsible gambling (RG) framework, mainly shaped by the Reno model approach, that emphasizes voluntary harm-reduction measures while avoiding interference with recreational players [7]. The model places emphasis on individual responsibility, promoting informed choice, personal control, and prioritization of gambling recreational values. In practice, this framing appears limited, since responsible gambling measures tend to be underutilized and insufficiently understood by many gamblers [8]. A growing body of literature points to an emerging shift towards a public health approach. This is largely driven by regulatory reforms that enforce public policy and governmental legislation to regulate gamblers' behaviour in such a way as to reduce the likelihood of gambling-related harm, including duty of care obligations aimed at protecting players from gambling-related harm [9]. Duty of care encompasses several key components, including player monitoring, proactive customer engagement, escalating interventions based on gambling severity, and player education [10, 11]. However, the persistent underutilization of RG tools, combined with the evolving expectations of RG, highlights the need for more comprehensive and proactive measures to support individuals who gamble.

An important yet often overlooked dimension of the duty of care involves ensuring that operators proactively equip customers with the information, tools, and resources required to make informed, ongoing decisions and to prevent harm. This involves frequent information on spending behaviour as well as addressing misconceptions or irrational beliefs about gambling outcomes. It has been hypothesized that providing information to encourage behavioural change can foster self-reflection and thus more RG practices [12]. Nevertheless, there is a dearth of knowledge in terms of the effectiveness of RG measures targeting erroneous beliefs, their implementation approaches, and their impact on online gambling. Against this backdrop, the current study evaluates an integrated educational intervention designed to correct potential erroneous gambling beliefs in a real-world online gambling setting.

Erroneous beliefs in gambling constitute irrational perceptions about gambling outcomes such as illusions of one’s self-control, misunderstanding probabilities, or believing that random outcomes from the past influence future wins (gambler's fallacy), which can lead to risky gambling behaviour and poor spending decision-making. Erroneous beliefs have been a subject of discussion from different perspectives. For example, Armstrong et al. [13] argued that the underlying mechanism behind an individual’s cognitive decision-making process plays a significant role in irrational gambling behaviour. Based on the dual process theory, they argue that gamblers often rely on intuitive assumptions drawn from their surroundings to make sense of the gambling environment, which leads to a biased interpretation of how gambling works. Erroneous gambling-specific beliefs such as misunderstandings about the concept of chance and the independence of consecutive draws are also identified as the aetiology of disordered gambling [14, 15].

Studies on efforts to educate gamblers have, so far, yielded contrasting results. Ferland [16] reported a positive result from an experiment involving young gamblers, in which a video-based intervention significantly improved participants’ understanding of gambling and helped correct their misconceptions about gambling products. Other intervention formats have been explored educating gamblers and addressing erroneous beliefs by providing information about odds, randomness, and probabilities in gambling. For example, educational programs offered at information centers in land-based casinos have been shown to modify misconceptions about randomness, but not gambling behaviour [17]. In another land-based casino setting, Louderback et al. [18] evaluated a multi-year implementation of the GameSense RG program and found that although greater awareness and engagement were linked to increased use of RG strategies, these effects did not reliably translate into improved gambling literacy or meaningful behavioural impact. All reported effects were weak, underscoring the practical limitations of educational RG efforts even in structured land-based environments.

Similarly, animated videos explaining how slot machines work have demonstrated effectiveness in correcting misconceptions [19] while efforts to educate young gamblers about gambling and probability have shown no significant effect [20]. In one school-based study, an intervention aiming to correct cognitive distortions (gambler’s fallacy and superstitious thinking) worked better than a control condition in reducing cognitive distortions [21]. In a simulated EGM study using credits, Monaghan et al. [22] found that participants who experienced losses exhibited a greater reduction in erroneous beliefs than those who experienced wins; however, the study did not control for factors such as gender or prior EGM familiarity. These studies highlight the diverse approaches taken to promote informed gambling behaviour, the consequences of gambling activities, and individual experiences associated with cognitive biases.

Not all products of gambling, however, are equally associated with problem gambling [23]. Due to its structural design and format, casino gambling is more strongly associated with problem gambling than other forms of gambling [24]. Studies focusing on understanding the underlying mechanisms of erroneous beliefs tend to examine individual cognitive factors such as gambling fallacy endorsement, general intelligence, reasoning ability, and rationality style, rather than how the design of gambling products can create cognitive biases [25]. This individual-centric focus seems to align with the field’s emphasis on personal responsibility over industry obligations. However, research on how product design and marketing, particularly in the form of customer experience, can contribute to cognitive errors is scarce [26]. This underscores the need to consider how game features, such as near misses [27] and losses disguised as wins [28], can influence gambling behaviour.

Compared to land-based settings, online gambling environments are typically more immersive, fast-paced, and privately accessed. These structural features may reduce the cognitive salience of brief educational interventions and limit opportunities for reflection. Despite growing interest in an online RG intervention, few studies have examined educational efforts specifically targeting erroneous beliefs in online gambling environments. In one such study, Auer and Griffiths [29] tested belief-based feedback in an online setting, reporting modest effects on gambling intensity. Their research has also demonstrated that self-monitoring is a key factor in guiding behaviour toward desired outcomes. In the same vein, Gainsbury et al. [30] used focus groups to examine preferences for responsible gambling messages and found that while tailored content was perceived positively across different gambler profiles, this did not reliably prompt uptake of harm reduction tools. These findings underscore the lack of large-scale, field-based studies examining whether belief-targeting educational interventions can meaningfully influence player behaviour.

The impact of different intervention formats (e.g., mandatory versus voluntary) has also been widely discussed in studies, particularly in contexts such as self-tests and pre-limit settings [10, 31, 32]. Nevertheless, we found limited literature examining the delivery of educational resources to players in real-life gambling settings under mandatory versus voluntary conditions. Most of the studies on the topic utilize experimental designs, where the experimental group is exposed to educational pop-up messages [33], while control groups receive no such exposure. In one field study [34], where the effects of a warning banner were used to inform the randomness of Video Lottery Terminals, outcomes revealed a significant decrease in faulty gambling beliefs among problem gamblers and an overall decrease in gambling behaviour. These experiments demonstrate that even in a non-mandatory setting, exposure to education about irrational beliefs in gambling outcomes can positively influence gambling behaviour.

Finally, we have also identified studies examining how various educational formats and timing influence the effectiveness of educational efforts in correcting erroneous gambling beliefs. Wohl et al. [19], for instance, found that animation-based education is more effective than controlled videos in an experiment that involved randomly assigned slot machine venues. In an Electronic Gaming Machine (EGM) experiment, a study [35] found that participants who watched an educational animation explaining how EGMs work were more likely to stick to their preset limits compared to those who did not receive the education. While the online gambling environment presents many opportunities to correct erroneous beliefs, interventions often prioritize on-screen pop-up warning messages and reminders, such as displaying money and time spent, rather than using educational resources as a direct intervention tool [36]. When educational formats are incorporated into online gambling, they are typically presented passively on operators’ RG pages [37]. The implementation of these efforts has not been successful [8, 38, 39]. Also, overall, these measures are viewed as positive for most individuals who gamble [40], but some have reacted to certain aspects in a negative way [41]. Importantly, there remains a notable lack of real-world evaluations of educational interventions specifically targeting erroneous gambling beliefs in online gambling platforms. Most existing studies have been conducted in either laboratory or land-based settings, underscoring the need for field-based research, including online gambling environments [42].

Addressing this gap, the current study aimed to evaluate the implementation and effectiveness of educational interventions in correcting erroneous gambling beliefs in a naturalistic online gambling environment. Specifically, we investigated whether the effectiveness of education-based interventions is impacted by factors related to how the intervention is presented to players, including the type (video lesson versus interactive message), timing (pre-game versus during gambling), and format (mandatory versus voluntary) of the intervention. Focusing on new online casino players, we also evaluate how different combinations of these intervention settings influence post-gambling behaviour and the use of RG measures.

### Objective and hypotheses

We examine the effect of educational interventions on erroneous beliefs implemented in the online gambling setting at the Norsk Tipping gambling platform for online casino customers in Norway. This experimental study included a non-intervention control group, and we compared the video and quiz presentations, voluntary and mandatory formats, and the timing of the intervention (before and during play).

While previous studies have shown mixed results regarding the behavioural effects of educational interventions, some empirical patterns inform our hypotheses. Video-based formats, for example, have been associated with greater comprehension and user engagement [19, 30], while mandatory exposure has consistently been linked to higher uptake and completion [31, 32]. Although quiz-style interventions are less studied, they may support self-monitoring by prompting active reflection [29]. Based on these design insights and operational considerations, we formulated directional hypotheses reflecting how such interventions are intended to function in practice, rather than as strong predictions derived from a consistent evidence base. For this study, we proposed the following hypotheses:

#### H1

 The video intervention (regardless of the exposure method^1^) is expected to be more effective than the control condition. Specifically, we anticipate less gambling activity (H_1a_) and greater use of RG measures (H_1b_) in the intervention condition compared to the control condition.

#### H2

 The Quiz intervention (regardless of exposure methods) will be more effective than the control condition. More specifically, we anticipate less gambling activity (H_2a_) and a greater use of RG measures (H_2b_) in the intervention condition compared to the control condition.

#### H3

The mandatory pregame intervention will be more effective than the voluntary pregame intervention. Specifically, we anticipate less gambling activity (H_3a_) and greater use of RG measures (H_3b_) in the mandatory condition compared to the voluntary condition.

## Method

### Setting

Norsk Tipping, a Norwegian state-owned gambling company, offers a portfolio of games, including retail and online lotteries, sports betting, online bingo, online casino games, and video lottery terminals (VLTs) at land-based venues. During the data collection period, the Norsk Tipping covered approximately 78% of the regulated gambling market in Norway, and 67% of the total market in terms of gambling revenue, encompassing all forms of regulated gambling except horse racing and bingo held at physical locations [43]. In principle, all gambling activities at Norsk Tipping, except physical scratch tickets, are registered at the individual level in order to enforce various forms of RG tools. These data also provide a strong foundation for monitoring the impact of various interventions. This experiment is part of a larger project at Norsk Tipping called *Spillepuls* (“Gambling Pulse” in English), in which more than 20 different real-time online intervention experiments were conducted.

### Design and participants

All data was already collected by Norsk Tipping as part of their everyday operations. For this study, we used the Norsk Tipping data warehouse as our data source.

**Fig. 1:**
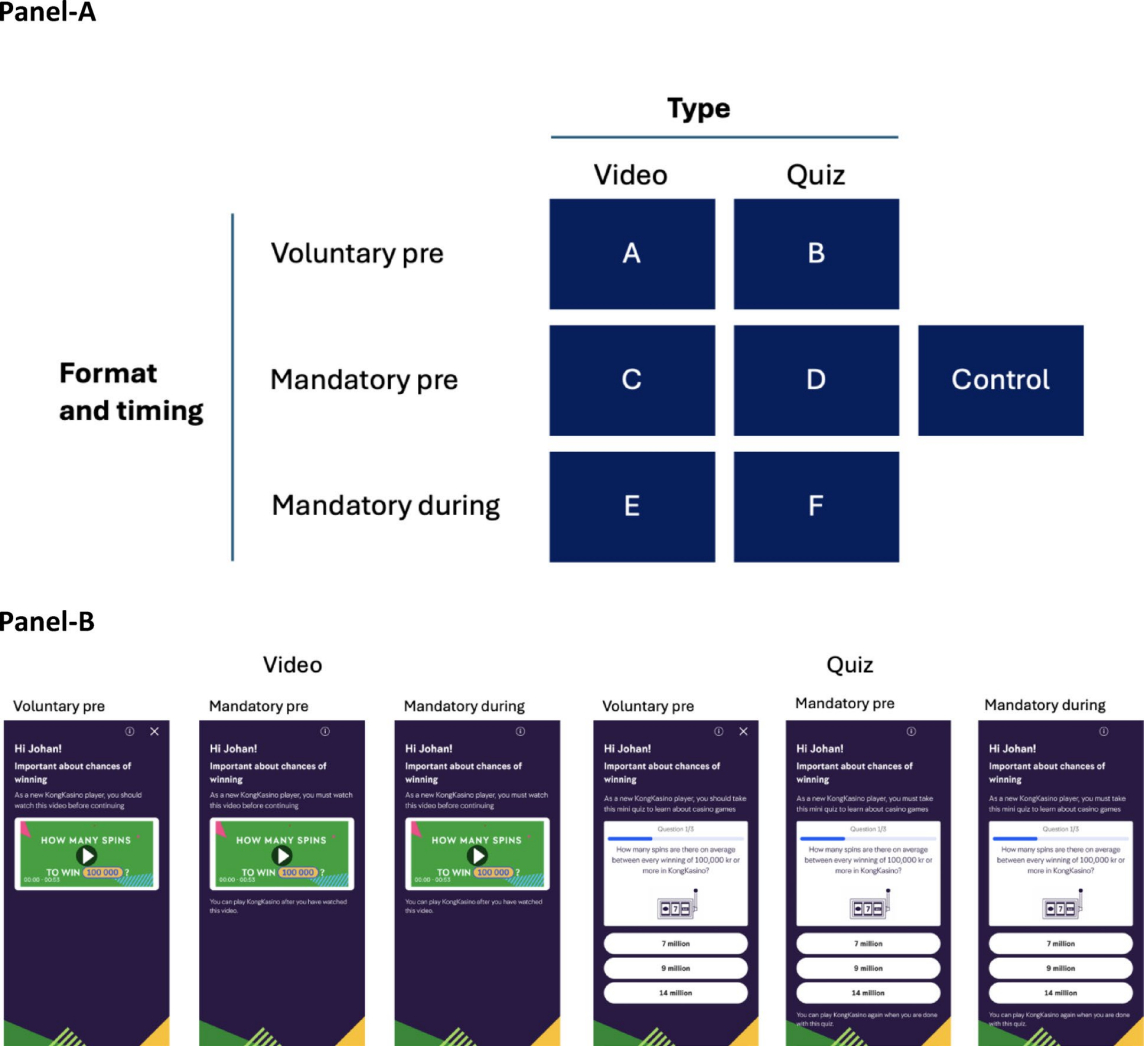
Distribution of exposures. Panel A shows the intervention design. Panel B shows how the interventions looked on the customers’ mobile screens

For this experiment, Norsk Tipping randomly assigned 18 981 new online casino players, referred to as customers in our study, on their interaction on the third day of play, to one of three treatment conditions: Video (3/7 allocation), Quiz (3/7 allocation), or a control group (1/7 allocation), between May 9th, 2022, and January 1st, 2023. Customers in the Video and Quiz groups were further randomly assigned to receive the intervention at one of three levels: voluntary before gameplay, mandatory before gameplay, and mandatory during gameplay (Fig. 1, Panel A). Sample sizes for the seven groups are found in Table 1.

**Table 1:** Baseline characteristics and customer engagement: proportion of participants who started and completed the intervention within each intervention group

Interventions and timings	Sex % women	Age (SD)	Number of customers started	Number of customers Finished	
				Of started	Of exposed
Video, voluntary pre-game (n = 2 720)	27.2	31.4 (14.0)	1 153/2 720 42.4%	194/1 153 16.84%	194/2 720 7.1%
Quiz, voluntary pre-game (n = 2 715)	27.6	31.6 (14.3)	1 061/2 715 39.1%	642/1 061 60.5%	642/2 715 23.6%
Video, mandatory pre-game (n = 2697)	26.7	31.6 (14.2)	2 693/2 697 99.8%	2 645/2 693 98.2%	2 645/2 697 98.1%
Quiz, mandatory pre-game (n = 2 712)	29.0	32.0 (14.3)	2 710/2 712 99.9%	2 689/2 710 99.2%	2 689/2 712 99.2%
Video, mandatory during game (n = 2 628)	27.2	31.3 (14.3)	2 415/2 628 91.9%	1 827/2 415 75.6%	1 827/2 628 69.5%
Quiz, mandatory during game (n = 2 679)	26.9	31.3 (13.8)	2 471/2 679 92.2%	2 225/2 471 90.0%	2 225/2 679 83.0%
Control group (n = 2 830)	27.5	31.6 (14.4)			

Of started = customers who started the intervention. Of exposed = customers who received the message. SD standard deviation

Participants were randomly assigned to one of the intervention groups. Two digital interventions, a *Video* and a *Quiz*, were presented on participants’ screens as pop-up messages on their third day of online casino interaction. Those in the intervention arms were further randomized into one of three viewing conditions as follows:

- *Voluntary pre-gameplay* (A and B): Was displayed when the customer logged into the casino lobby before starting to play. The message appeared up to three times if the customer did not respond to it.
- *Mandatory pre-gameplay* (C and D): Was shown when the customer entered the casino lobby at the start of the game. It appeared every time the customer attempted to open a game until the intervention was attended.
- *Mandatory during gameplay* (E and F): The pop-up appeared after the customer closed their first game and continued to be displayed each time they attempted to open a new game until the intervention was responded to.

The time required to complete the Video intervention was 49 s, while the Quiz intervention consisted of three questions covering the same topics as the Video. The Quiz questions were:

1. How many spins are there between each prize of NOK 10,000 or more? (Multiple choice with only one option as correct). (Fig. 1, Panel B).
2. If you lose several times in a row, does the probability of winning increase next time? (Yes/No).
3. How many winnings in casinos are negative? Multiple choice with only one option as correct.

### Blinding

The study was single-blind, implying that the participants were not informed that they had been randomly assigned to one of the three conditions.

### Pre-registration

The study, including hypotheses, was pre-registered [44].

### Ethical statement

The study setup and data collection were conducted as part of Norsk Tipping’s everyday operations within the Spillepuls project. The last author was consulted regarding the study setup and design. The researchers independently determined the outcome measures and had full scientific freedom. The Swedish Ethical Review Authority approved the transfer of personal data for an independent scientific evaluation (2024-01578-01). Karolinska Institute was awarded a contract for commissioned research on responsible gambling through an open tender in 2022, which financed the first author’s work.

### Measures and data collection

#### Outcomes measurement

Each outcome was compiled from the operator's data warehouse, and made available to the researchers in raw format as weekly aggregates over 24 weeks: 12 weeks before the intervention phase and 12 weeks following the intervention (post-phase). *Gambling behaviour* was measured as the sum and number of wagers, theoretical loss and net loss – for all games and for casino games respectively. Theoretical loss is defined as total bet x house advantage, which is (bet—((bet x payback %) / 100)). Theoretical loss is regarded as the most stable measure of gambling intensity [45]. Payment behaviour was measured as the number of deposits and the sum of deposits and the net deposits^2^ in Norwegian kroner (NOK) to and from the customers’ gambling accounts.

*The use of RG measures* was evaluated by assessing the number of self-tests performed, breaks/pauses and self-exclusions taken, and loss-limit changes; for the purpose of analyses, counts were aggregated over the full post-phase and dichotomized (used or not).

*Rating of intervention.* At the end of the intervention, the participants were asked to rate the intervention’s a) relevance, b) usefulness, and c) disruptance on a scale of 1 (disagree) to 5 (agree). Post-hoc analyses on the gambling measures described above revealed that those who did not provide intervention ratings gambled less than those who did, pre-intervention (week –1), see Supplementary material Table S1.

### Statistical analysis

The present study examined the impact of two intervention types (Video and Quiz), in voluntary and mandatory formats, and with two different viewing conditions (pre- or during gameplay), compared to a control condition, on gambling behaviour and the use of RG measures. Since gambling data with full population coverage often show long tails and since gambling activity is often periodical (resulting in weekly aggregates with no recorded gambling activity, i.e., excess zeroes), we opted to use quantile regression (with bootstrapped confidence intervals) to analyse post-intervention gambling behaviour outcomes across study arms. Importantly, quantile regression makes no assumption about the underlying distribution and is therefore robust to outliers. Primary analyses were conducted in two steps: first, first-week post-intervention outcomes across any intervention versus control were compared; next, first-week post-intervention outcomes were compared across intervention arms (control group excluded) using fully factorial models (format × viewing option). Since all participants were new casino gamblers (i.e., no pre-intervention casino activity available), and other gambling types are highly periodic in nature, no adjustment for pre-intervention gambling was included in the models. Although longitudinal quantile regression modelling was considered, this was dropped in lieu of hypotheses about the longitudinal trajectories of any effects. First-week outcomes were chosen since there were no a priori estimates of the durations of any effects, since longer aggregates risk obfuscating short-term effects. However, due to the high proportion of true zeros, we were not able to obtain reliable first-week post-intervention estimates for neither the 0.5 and 0.75 quantiles on the outcomes total number of deposits or the total sum of deposits; hence, for these variables, we aggregated these respective outcomes over the entire 12-week post-intervention. To aid interpretation, we also performed secondary analyses on the remaining outcomes using the same 12-week post-intervention aggregates, the results of which are presented in the supplementary material. To examine the impact on use of RG tools, and whether there were significant differences in adherence to the interventions between the groups, Chi-square tests were used.

## Results

### Adherence

Table 1 describes the customers’ engagement across six intervention groups, showing the proportion of customers who started and finished the interventions. The highest engagement and completion rates were not unexpectedly observed for the mandatory interventions, particularly for the mandatory pre-game quiz intervention, where almost all customers completed the intervention. In contrast, voluntary interventions showed lower participation rates, with the lowest completion rates observed for the voluntary video pre-game intervention, Chi^2^ (1, N = 5,435) = 283.4, *p* < 0.001). Consequently, mandatory interventions had higher completion rates than voluntary interventions, Chi^2^ (1, N = 12 503) = 3 492, *p* < 0.001. Among the voluntary interventions, the quiz format had a notably higher completion rate than the video format, Chi^2^ (1, N = 2,214) = 448.6, *p* < 0.001, indicating that customers who chose to engage with quizzes were more likely to complete them compared to those who started the videos.

Within the mandatory conditions, pre-game interventions had the highest completion rate (99%), with a small but significant difference between video and quiz formats (99.8% versus 98.2%, Chi^2^ (1, N = 5 403) = 10.9, *p* < 0.01). In contrast, mandatory during-game interventions had lower completion rates than mandatory pregame interventions (83.0% versus 99.2%, Chi^2^ (1, N = 10 289) = 799, *p* < 0.001). Still, the completion rate was higher in the quiz condition (90.0%) compared to the video format (75.6%) among those in the mandatory during game intervention (Chi^2^ (1, N = 4 886) = 179, *p* < 0.001).

These findings highlight that mandatory delivery, particularly pre-game, was the most effective method to ensure intervention completion, while voluntary engagement had the lowest adherence for both quiz and video.

### Rating of intervention

Table 2 provides an overview of customers' ratings of the interventions across dimensions such as relevance, usefulness, and disruptance. Overall, video interventions received higher average ratings for both relevance and usefulness compared to quiz interventions, while quiz interventions were generally rated as less disrupting on average. However, it is important to note that the proportion of participants who rated any intervention as disrupting was relatively low, particularly for voluntary video interventions, where only 24.7% of respondents reported disruption (n = 46). In contrast, quiz interventions and mandatory delivery, particularly during the game, were less frequently rated as disrupting and also showed lower average disruption scores.

**Table 2:** Rating of the different interventions by the customers on relevance, usefulness, and disruptance. Scale 1 (disagree) to 5 (agree)

Interventions	Rated /Finished intervention (%) Relevance	Relevance Mean (SD) (95%CI)	Rated /Finished intervention (%) Usefulness	Usefulness Mean (SD) (95%CI)	Rated /Finished intervention (%) Disruptance	Disruptance Mean (SD) (95%CI)
Video, voluntary pre-game	46/194 = 23.7	4.54 (0.88) (4.29, 4.79)	46/194 = 23.7%	4.45 (0.86) (4.20, 4.70)	46/194 = 23.7%	4.43 (0.88) (4.18, 4.68)
Quiz, voluntary pre-game	244/642 = 38.0	4.14 (1.06) (4.01, 4.27)	243/643 = 37.8%	4.08 (1.06) (3.95, 4.21)	230/642 = 35.8%	4.12 (1.07) (3.98, 4.26)
Video, mandatory pre-game	1114/2645 = 42.1	4.06 (1.06) (4.00, 4.12)	1 096/2 645 = 41.4%	4.04 (1.06) (3.98, 4.10)	1 090/2 645 = 41.2%	4.02 (1.09) (3.96, 4.04)
Quiz, mandatory pre-game	976/2689 = 36.3	3.70 (1.22) (3.62, 3.78)	943/2 689 = 35.1%	3.70 (1.22) (3.62, 3.78)	919/2 689 = 34.2%	3.69 (1.25) (3.61, 3.77)
Video, mandatory during game	620/1827 = 33.9	4.16 (1.05) (4.08, 4.24)	632/1 827 = 34.6%	4.09 (1.08) (4.01, 4.17)	616/1 827 = 33.7%	4.10 (1.09) (4.01, 4.19)
Quiz, mandatory during game	848/2225 = 38.1	3.81 (1.17) (3.73, 3.89)	832/2 225 = 37.4%	3.76 (1.18) (3.68, 3.84)	822/2 225 = 36.9%	3.73 (1.21) (3.65, 3.81)

SD standard deviation; CI confidence interval. Statistical significance can be inferred from comparing confidence intervals

Video or quiz delivery was perceived as more relevant and useful in the voluntary intervention compared to the mandatory one. Among the mandatory interventions, those delivered pre-game were rated slightly lower in relevance and showed almost no difference in terms of usefulness compared to those delivered during the game. While they were also perceived as less disrupting on average, this pattern should be interpreted with caution, given the low number of participants who reported disruptance in certain conditions.

#### Gambling and RG outcomes

As depicted in Table 3, there were no differences between the intervention groups and the control group regarding gambling behaviour. See supplementary Table S2 for results aggregated over the full post period. There were also no difference regarding the use of RG measures post-intervention, performed self-tests video versus control (Chi^2^ (1, N = 10,875) = 0.13, *p* 0.714), quiz versus control (Chi^2^ (1, N = 10,936) = 1.66, *p* 0.197), breaks/pauses video versus control (Chi^2^ (1, N = 10,875) = 1.56, *p* 0.211), quiz versus control (Chi^2^ (1, N = 10,936) = 1.96, *p* 0.161) self-exclusions video versus control (Chi^2^ (1, N = 10,875) = 1.04, *p* 0.309), quiz versus control (Chi^2^ (1, N = 10,936) = 0.14 *p* 0.704) and loss-limit changes video versus control (1, N = 10,875) = 2.45, *p* 0.086), quiz versus control (Chi^2^ (1, N = 10,936) = 0.85 *p* 0.356). Thus, H1a, H1b, H2a, and H2b were not supported. However, partially lending support for H3a, mandatory pre-game interventions were associated with lower overall theoretical loss, overall total sum of wagers, and number of deposits, as well as lower theoretical loss for casino games, compared to voluntary pre-game interventions. A significant interaction between intervention format and mandatory pre-game delivery was also observed for overall theoretical loss. This suggests that the effect of mandatory pre-game delivery differed between the video and quiz formats. Although several first-week differences become less apparent when gambling behaviour was aggregated across a full follow up, some interaction effects remained evident (see Table S2). For other outcomes (overall number of wagers, sum of deposits, number of wagers of casino games, and sum of wagers of casino games) no differential effects were found (see Table 3). H3b was not supported, there were no differences in use of RG measures between voluntary and mandatory pre-game interventions, performed self-tests (Chi^2^ (1, N = 10,844) = 0.07, *p* 0.787), breaks/pauses (Chi^2^ (1, N = 10,844) = 1.06, *p* 0.303), self-exclusions (Chi^2^ (1, N = 10,844) = 0.06, *p* 0.806) and loss-limit changes video versus control (1, N = 10,844) = 0.01, *p* 0.908).

**Table 3:** Quantile regression outcomes

Outcome	Model 1		Model 2					
	Intercept β 95% CI	Any intervention β 95% CI	Intercept (REF-Quiz, REF-Voluntary pre) β 95% CI	Type (REF-Quiz vs Video) β 95% CI	Viewing condition (REF-Voluntary pre vs Mandatory pre) β 95% CI	Viewing condition (REF-Voluntary pre vs Mandatory during) β 95% CI	Video*Mandatory pre β 95% CI	Video*Mandatory during β 95% CI
Number of wagers at the casino (75th percentile)	419 (368.84, 469.16)*	−5 (−56.95, 46.95)	434.00 (379.77, 488.23)*	−38.00 (−103.44, 27.44)	−29.00 (−97.14, 39.14)	22.00 (−48.48, 92.48)	47.00 (−51.14, 145.14)	−34.00 (−121.47, 53.47)
Sum of wagers at casino (75th percentile)	2570 (2302.60, 2837.40)*	−62 (−350.37, 226.37)	2689.00 (2353.85, 3024.15)*	−207.60 (−619.15, 203.95)	−336.60 (−722.35, 49.15)	−6.50 (−424.61, 411.61)	322.20 (−187.06, 831.46)	−59.90 (−601.94, 482.14)
Sum of TL casino (75th percentile)	21.95 (19.54, 24.36)*	0.42 (−2.14, 2.98)	22.96 (20.24, 25.68)*	−1.05 (−4.55, 2.46)	−3.95 (−7.72, −0.18)*	3.98 (−0.20, 8.15)	3.11 (−2.11, 8.33)	−3.63 (−9.42, 2.16)
Number of wagers at total gambling (50th percentile)	110 (100.00, 120.00)*	−2 (−13.09, 9.09)	108.00 (94.14, 121.86)*	2.00 (−14.54, 18.54)	−7.00 (−24.70, 10.70)	18.00 (−0.77, 36.77)	−3.00 (−24.19, 18.19)	−21.00 (−43.70, 1.70)
Sum of wagers at total gambling (50th percentile)	830 (766.12, 893.88)*	−15 (−56.81, 86.81)	875.00 (795.64, 954.36)*	−32.50 (−147.63, 82.63)	−115.00 (−208.57, −21.43)*	67.30 (−57.67, 192.27)	91.50 (−50.56, 233.56)	−57.80 (−216.30, 100.70)
TL total gambling (50th percentile)	83.60 (76.43, 90.77)*	−2.17 (−9.79, 5.45)	86.10 (79.83, 92.37)*	−8.22 (−18.61, 2.17)	−9.96 (−18.12, −1.81)*	−1.92 (−11.56, 7.71)	14.32 (0.99, 27.65)*	4.06 (−9.76, 17.88)
Number of deposits^x^ (75th percentile)	3 (2.40, 3.60)*	0 (−0.60, 0.60)	3.00 (2.86, 3.14)*	0.00 (−0.83, 0.83)	−1.00 (−1.89, −0.11)*	−0.00 (−0.41, 0.41)	1.00 (−0.32, 2.32)	0.00 (−0.97, 0.97)
Sum of deposits^x^ (75th percentile)	1837.15 (1556.62, 2117.68)*	−165.27 (−478.46, 147.92)	1600.55 (1315.26, 1885.84)*	−0.55 (−341.67, 340.57)	−95.20 (−479.66, 289.26)	196.45 (−201.73, 594.63)	187.65 (−317.35, 692.65)	−3.45 (−561.97, 555.07)

CI confidence interval; TL theoretical loss. ^x^Aggregated across the entire post-period, otherwise first-week post-intervention. *Statistical significance. Statistical significance can be inferred from whether the confidence intervals include zero

## Discussion

Our aim was to examine how the effectiveness of educational interventions among new casino players varied based on their type (video versus interactive quiz), timing (pre-gambling versus during gambling), and format (mandatory versus voluntary). Additionally, independent of their effectiveness, the setting enabled us to observe participants’ engagement with the intervention and rating of the interventions’ formats. The study was conducted on the online casino platform of a gambling operator, specifically targeting new online casino players.

The overall findings show no sustained effect of any intervention type on gambling behaviour, nor any uptake in the use of RG-measures. This was observed across examined indicators and for all RG tools assessed. The finding is consistent with previous research suggesting that correcting erroneous gambling beliefs does not necessarily translate into long-term behavioural change [46]. In partial support of H3a, when comparing intervention arms, mandatory pregame delivery was associated with a short-lived effect with lower overall theoretical loss, overall total sum of wagers, and number of deposits, as well as lower theoretical loss for casino games, relative to voluntary pregame delivery. Comparison with the aggregated analyses indicated that this pattern was not consistently reproduced across the full follow-up period, although certain main and interaction effects remained statistically significant. In contrast, several other indicators (overall number of wagers, sum of deposits, number of wagers of casino games, and sum of wagers of casino games) showed no corresponding differences. In addition, no differences were observed between these conditions in the uptake of RG measures, and thus H3b was not supported. These findings indicate that while delivery mode may influence certain monetary measures, the overall pattern suggests no sustained influence on gambling behaviour. Moreover, comparison with the aggregated analyses (Table S2) showed that not all first-week findings were reproduced across outcomes, which warrants cautious interpretation of the observed effects.

Based on participant ratings (Table 2), video interventions were perceived as more relevant and useful among participants who completed the study. In contrast, quiz formats achieved higher completion rates, particularly when delivered voluntarily. This divergence shows perceived relevance does not necessarily translate into higher engagement. In addition, we observed that mandatory pregame delivery resulted in consistently high exposure across formats, whereas voluntary engagement remained substantially lower, particularly for the video content. The observed contrast reflects that while delivery mode primarily shapes exposure, format influences perceived usefulness, without necessarily affecting behavioural outcomes.

Consistent with similar findings examining other forms of gambling (i.e., slot machines) [47], we observed that short videos that combine audio and visual messages may enhance perceived clarity or memorability. This finding also aligns with the seminal work of Paivio’s Dual Coding Theory [48], which posits that using both verbal and visual channels enhance learning more effectively than text-based instructions. Even though audiovisual formats may enhance perceived salience, the present findings indicate that brief exposure alone is insufficient to produce measurable behavioural change. Future research should therefore examine how visual or interactive elements can be embedded within more sustained RG strategies. For instance, structured onboarding procedures or product-specific educational components may provide repeated opportunities for reflection prior to gambling.

A closer examination of timing and delivery reveals clear differences in engagement. For instance, completion rates were highest when the intervention was mandatory and delivered before gambling began, whereas engagement declined substantially when delivery was voluntary or occurred during gameplay. In line with current literature [32], interventions delivered during play may be perceived as disruptive, which could contribute to lower completion rates. The results demonstrate that timing can influence exposure and user receptivity, and that the placement of interventions within the gambling flow shapes engagement, regardless of measurable behavioural effects.

This pattern was particularly evident for quiz-style interventions. Participants rated quizzes as less intrusive than video-based formats, including when delivered during gameplay. Quizzes also achieved higher completion rates under voluntary delivery. As such, interactive formats may be more compatible with the gambling context in terms of perceived disruption and engagement. However, this advantage did not also translate into measurable behavioural differences.

Aligned with earlier research on mandatory delivery formats [49], mandatory interventions in the present study resulted in substantially higher completion rates across both video and quiz formats. Completion was particularly high when delivery occurred before gambling began. In contrast, voluntary interventions showed markedly lower uptake. Participants also rated voluntary interventions as more relevant but more disrupting than mandatory ones. The pattern points to a tension between autonomy and exposure in the delivery of educational content. While voluntary participation may reflect higher perceived relevance among those who choose to engage, it is associated with lower overall reach. Conversely, mandatory delivery ensures broad exposure but may reduce perceived personal relevance. As such, delivery format can shape both reach and user evaluation, even if it does not translate into measurable behavioural change.

One implication of our study is the need for a multidisciplinary approach to developing effective methods for communicating educational interventions. While the design of RG tools (e.g., nudging interfaces) has been informed by behavioural economics and human–computer interaction research [50], comparatively less attention has been paid to insights from educational and learning sciences. Research on visual learning, for example, suggests that multimodal formats may influence comprehension and recall differently than text-based approaches [51–54]. In light of the present study, future research should examine how such perspectives can inform the structure, sequencing, and repetition of digital RG interventions.

The findings also point to the potential value of more adaptive intervention strategies. Rather than relying on uniform delivery formats, future research could examine whether tailoring educational content to different player profiles or gambling contexts affects acceptability and engagement. The observed contrast between high exposure under mandatory delivery and higher perceived relevance under voluntary conditions underscores the need to balance reach with user experience. Future research should examine how intervention designs can achieve broad exposure while minimizing perceived disruption during gameplay. For instance, greater attention to user-centred investigation may help clarify how players engage with RG content in practice. Observational approaches – e.g., think-aloud protocols [55] or interaction-tracking methods [56] – could provide insight into how individuals navigate and interpret interventions within real gambling environments. Such perspectives may inform the development of more context-sensitive intervention designs.

A key strength of the study is its randomized controlled design, which enhances internal validity by minimizing selection bias and enabling direct comparisons across intervention conditions. The trial was embedded within a real-world gambling platform, supporting ecological relevance. In addition, the use of objective behavioural data from a large sample of online casino players strengthens the reliability of the findings within the studied context. The methodological approach may inform future research examining heterogeneous responses across gambler profiles and support replication in comparable real-world settings.

The study has several limitations. First, we did not directly assess whether the video or quiz interventions altered participants’ erroneous gambling beliefs. As such, we cannot determine whether the absence of behavioural effects reflects limited cognitive change or a disconnect between belief modification and behavioural outcomes. In addition, the sample consisted of new online casino customers, restricting the pre-intervention observation window. Although the analytic choice of comparing outcomes at different quantiles does offer some indirect insight into how effects differ across gambling activity levels, analyses were not directly stratified by risk level; future research should examine whether responses vary across distinct player profiles.

Second, the study was conducted within a regulated operator context characterized by bet restrictions and mandatory spending limits. These structural safeguards may have constrained behavioural variability and moderated potential intervention effects. Furthermore, only a subset of participants provided intervention ratings, and non-responders exhibited lower gambling activity than responders, which may limit the interpretability of subjective evaluations. Replication across diverse regulatory and operator contexts would clarify the generalizability of these findings.

Finally, interventions targeting individual-level cognitive distortions, while scalable and easy to implement, may risk emphasizing individual responsibility over systemic approaches to harm prevention. The present findings should therefore be understood as an evaluation of one specific component within a broader responsible gambling ecosystem. While no meaningful behavioural effects were observed, the results provide insight into how different educational formats are received and engaged with by users. Specifically, the preference for video-based content, alongside the higher completion rates of interactive formats, suggests that these tools could be preferable as complementary components designed to foster awareness and engagement rather than as primary drivers of behavioural change. When integrated into a public health-oriented, multi-level strategy, such interventions may serve as a low-threshold entry point for harm reduction [57]; however, their impact is likely to remain modest unless implemented in tandem with robust structural or product-level measures.

## Conclusions

Erroneous beliefs about gambling are widely recognized in the literature as a contributing factor to harmful gambling behaviour. Despite this, there is relatively little evidence regarding the effectiveness of digital intervention strategies designed to target these misconceptions in real-time online gambling environments. This study aimed to address this gap by evaluating the impact of educational interventions, delivered in different formats and under varying conditions, among a sample of new online casino customers.

Our findings suggest that mandatory interventions, particularly those delivered prior to gambling, achieved substantially higher completion rates. The voluntary formats were perceived by users as more relevant and more disrupting. However, neither delivery mode led to significant changes in subsequent gambling behaviour or increased use of RG tools. This indicates that brief educational prompts, as implemented in the present study, were insufficient to produce observable behavioural change.

Overall, the findings highlight the role of timing, format, and presentation in shaping users’ receptivity to RG messages. However, the absence of behavioural effects indicates that further refinement of educational content alone is unlikely to produce meaningful reductions in gambling harm. Educational interventions may still contribute within a broader RG framework, but their impact is likely to remain limited without complementary structural or product-level measures.

## Supplementary Information

Below is the link to the electronic supplementary material.

Supplementary Material 1

## Data Availability

Due to the nature of the research, due to commercial secrecy, supporting data is not available.

## Footnotes

1 The timing and if mandatory or voluntary.

2 Net deposit = sum of deposits minus sum of withdrawals.
