# Supplementary material for: Educational interventions for erroneous gambling beliefs among Norwegian online casino players: a large-scale real-world study of behavioural impact
Source: Harm Reduct J. 2026 Aug 6;23:127. doi: 10.1186/s12954-026-01508-9 (PMC13450470; doi:10.1186/s12954-026-01508-9)
Supplement: Supplementary file 1 [file 12954_2026_1508_MOESM1_ESM.docx]

Table S1. Quantile regression outcomes over full post-period – first model (any intervention vs control)

| **Outcome** | **Quantile** | **(Intercept)** | **Any intervention B [95% CI]** |
| --- | --- | --- | --- |
| N wagers | 0.25 | 114.00 [101.88, 126.12] | 2.00 [-11.13, 15.13] |
| N wagers - casino | 0.25 | 77.00 [63.73, 90.27] | -1.00 [-14.73, 12.73] |
| Nr deposits | 0.25 |  |  |
| Sum of deposits | 0.25 |  |  |
| Sum of TL | 0.25 | 187.68 [171.35, 204.00] | 0.63 [-17.59, 18.85] |
| Sum of TL - casino | 0.25 | 18.72 [16.34, 21.10] | -0.55 [-3.17, 2.08] |
| Sum of wagers | 0.25 | 1436.00 [1286.66, 1585.34] | 54.00 [-102.02, 210.02] |
| Sum of wagers - casino | 0.25 | 457.20 [393.71, 520.69] | -13.20 [-80.21, 53.81] |
| N wagers | 0.5 | 488.00 [441.41, 534.59] | 17.00 [-35.36, 69.36] |
| N wagers_casino | 0.5 | 419.00 [378.03, 459.97] | 2.00 [-42.78, 46.78] |
| Nr deposits | 0.5 | 0.00 [-0.97, 0.97] | -0.00 [-0.97, 0.97] |
| Sum of deposits | 0.5 | 0.00 [-55.38, 55.38] | -0.00 [-55.38, 55.38] |
| Sum of TL | 0.5 | 601.92 [549.68, 654.16] | -8.29 [-64.50, 47.93] |
| Sum of TL - casino | 0.5 | 110.08 [96.67, 123.50] | 1.15 [-13.16, 15.45] |
| Sum of wagers | 0.5 | 5159.00 [4752.94, 5565.06] | -152.00 [-589.78, 285.78] |
| Sum of wagers - casino | 0.5 | 2685.00 [2372.79, 2997.21] | 29.80 [-323.84, 383.44] |
| N wagers | 0.75 | 2381.00 [2148.96, 2613.04] | 71.00 [-189.23, 331.23] |
| N wagers_casino | 0.75 | 2213.00 [1957.84, 2468.16] | -12.00 [-282.44, 258.44] |
| Nr deposits | 0.75 | 3.00 [2.35, 3.65] | -0.00 [-0.67, 0.67] |
| Sum of deposits | 0.75 | 1837.15 [1576.22, 2098.08] | -165.27 [-458.80, 128.26] |
| Sum of TL | 0.75 | 1719.47 [1595.10, 1843.84] | -81.85 [-221.87, 58.18] |
| Sum of TL - casino | 0.75 | 574.09 [510.20, 637.97] | -23.52 [-94.47, 47.43] |
| Sum of wagers | 0.75 | 19362.00 [17258.98, 21465.02] | -665.20 [-2958.67, 1628.27] |
| Sum of wagers - casino | 0.75 | 14038.00 [12330.29, 15745.71] | -599.30 [-2493.58, 1294.98] |

Table S2. Quantile regression outcomes over full post-period – second, factorial model (control group excluded)

| Outcome | Quantile | (Intercept) | Type (Ref-Quiz vs Video) | Viewing condition (Ref-Voluntary pre vs Mandatory pre) | Viewing condition (Ref- Voluntary pre vs Mandatory during) | Video*Mandatory pre | Video*Mandatory during |
| --- | --- | --- | --- | --- | --- | --- | --- |
| Nr wagers | 0.25 | 124.00 [112.55, 135.45]* | 2.00 [-16.28, 20.28] | -19.00 [-32.51, -5.49]* | -0.00 [-19.23, 19.23] | -2.00 [-24.45, 20.45] | -7.00 [-34.58, 20.58] |
| Nr wagers - casino | 0.25 | 79.00 [67.21, 90.79]* | 4.00 [-11.56, 19.56] | -9.00 [-24.61, 6.61] | 2.00 [-12.94, 16.94] | -8.00 [-28.84, 12.84] | -7.00 [-27.89, 13.89] |
| Nr of deposits | 0.25 |  |  |  |  |  |  |
| Sum of deposits | 0.25 |  |  |  |  |  |  |
| Sum of TL | 0.25 | 188.30 [166.32, 210.29]* | 2.78 [-27.03, 32.59] | -10.05 [-37.74, 17.63] | 9.68 [-21.29, 40.65] | 0.24 [-39.91, 40.39] | -6.96 [-51.83, 37.90] |
| Sum of TL - casino | 0.25 | 19.23 [16.94, 21.52]* | -0.04 [-3.49, 3.41] | -3.98 [-7.21, -0.74]* | 1.97 [-1.86, 5.81] | 1.11 [-3.69, 5.91] | -2.52 [-8.04, 3.01] |
| Sum of wagers | 0.25 | 1547.40 [1382.71, 1712.09]* | -60.40 [-277.82, 157.02] | -73.20 [-277.90, 131.50] | 20.60 [-201.72, 242.92] | -17.40 [-278.69, 243.89] | 18.40 [-265.50, 302.30] |
| Sum of wagers - casino | 0.25 | 469.60 [416.89, 522.31]* | -1.60 [-83.95, 80.75] | -97.60 [-173.11, -22.09]* | 49.40 [-44.39, 143.19] | 28.60 [-82.53, 139.73] | -62.60 [-194.88, 69.68] |
| Nr wagers | 0.5 | 532.00 [463.44, 600.56]* | -40.00 [-120.43, 40.43] | -45.00 [-139.13, 49.13] | 41.00 [-54.68, 136.68] | 19.00 [-93.04, 131.04] | -51.00 [-167.50, 65.50] |
| Nr wagers - casino | 0.5 | 444.00 [387.16, 500.84]* | -29.00 [-101.83, 43.83] | -45.00 [-127.03, 37.03] | 45.00 [-36.88, 126.88] | 12.00 [-99.52, 123.52] | -63.00 [-159.84, 33.84] |
| Nr of deposits | 0.5 | 0.00 [-0.20, 0.20] | 0.00 [-0.46, 0.46] | -0.00 [-0.20, 0.20] | 0.00 [-0.89, 0.89] | 0.00 [-0.61, 0.61] | -0.00 [-1.03, 1.03] |
| Sum of deposits | 0.5 | 0.00 [-3.08, 3.08] | 0.00 [-14.41, 14.41] | -0.00 [-4.16, 4.16] | 0.00 [-43.08, 43.08] | 0.00 [-24.17, 24.17] | -0.00 [-54.20, 54.20] |
| Sum of TL | 0.5 | 637.72 [596.41, 679.03]* | -45.51 [-102.74, 11.72] | -84.34 [-144.84, -23.85]* | -64.82 [-131.83, 2.18] | 90.75 [4.33, 177.17]* | 77.35 [-11.15, 165.85] |
| Sum of TL - casino | 0.5 | 124.41 [112.40, 136.42]* | -15.68 [-32.85, 1.49] | -24.03 [-42.27, -5.79]* | -0.53 [-18.64, 17.59] | 16.91 [-9.37, 43.19] | -0.24 [-25.84, 25.35] |
| Sum of wagers | 0.5 | 5407.50 [4799.77, 6015.23]* | -450.50 [-1232.20, 331.20] | -730.30 [-1473.31, 12.71] | -95.50 [-802.72, 611.72] | 589.33 [-346.64, 1525.30] | 145.50 [-852.58, 1143.58] |
| Sum of wagers - casino | 0.5 | 3043.00 [2756.41, 3329.59]* | -392.00 [-781.96, -2.04]* | -594.75 [-1033.90, -155.60]* | -2.00 [-469.75, 465.75] | 425.60 [-147.98, 999.18] | -13.00 [-667.15, 641.15] |
| Nr wagers | 0.75 | 2402.00 [2161.98, 2642.02]* | -54.00 [-446.39, 338.39] | -17.00 [-419.38, 385.38] | 283.00 [-81.64, 647.64] | 185.00 [-445.43, 815.43] | -266.00 [-872.65, 340.65] |
| Nr wagers - casino | 0.75 | 2224.00 [2006.39, 2441.61]* | -206.00 [-559.71, 147.71] | -72.00 [-422.94, 278.94] | 202.00 [-106.41, 510.41] | 250.00 [-232.71, 732.71] | -84.00 [-534.05, 366.05] |
| Nr of deposits | 0.75 | 3.00 [2.66, 3.34]* | 0.00 [-0.92, 0.92] | -1.00 [-2.00, 0.00] | -0.00 [-0.57, 0.57] | 1.00 [-0.40, 2.40] | 0.00 [-0.97, 0.97] |
| Sum of deposits | 0.75 | 1600.55 [1325.03, 1876.07]* | -0.55 [-347.69, 346.59] | -95.20 [-454.73, 264.33] | 196.45 [-202.38, 595.28] | 187.65 [-331.16, 706.46] | -3.45 [-556.13, 549.23] |
| Sum of TL | 0.75 | 1720.00 [1578.62, 1861.39]* | -89.43 [-295.93, 117.07] | -177.54 [-378.54, 23.46] | -63.94 [-248.79, 120.91] | 195.25 [-94.06, 484.57] | 72.52 [-208.96, 354.00] |
| Sum of TL - casino | 0.75 | 607.65 [533.94, 681.36]* | -89.13 [-183.77, 5.51] | -101.46 [-197.52, -5.41]* | -47.46 [-154.66, 59.75] | 133.20 [-12.04, 278.45] | 101.21 [-33.60, 236.02] |
| Sum of wagers | 0.75 | 19483.50 [17392.19, 21574.81]* | -1860.55 [-4529.94, 808.84] | -2417.50 [-5289.48, 454.48] | -155.50 [-3247.35, 2936.35] | 4485.10 [602.38, 8367.82]* | 1391.55 [-2472.47, 5255.57] |
| Sum of wagers - casino | 0.75 | 14834.25 [13016.90, 16651.60]* | -2173.45 [-4523.05, 176.15] | -2461.69 [-4563.00, -360.38]* | -1170.95 [-3749.55, 1407.65] | 3221.89 [287.08, 6156.70]* | 2468.15 [-1046.02, 5982.32] |

TL Theoretical loss *Statistical significance. Statistical significance can be inferred from whether the confidence intervals include zero.
